# A bibliometric analysis and visualization of tension-type headache

**DOI:** 10.3389/fneur.2022.980096

**Published:** 2022-08-31

**Authors:** Xueming Fan, Guojing Fu, Liuding Wang, Wei Shen, Yunling Zhang

**Affiliations:** Xiyuan Hospital, China Academy of Chinese Medical Sciences, Beijing, China

**Keywords:** bibliometric analysis, CiteSpace, network, tension-type headache, VOSviewer

## Abstract

**Background:**

Tension-type headache (TTH) is the most prevalent headache in the clinical practice, leading to impaired social activities, work-related disability, and heavy financial burdens. Previous studies have described possible inducement, potential pathophysiology, and clinical management of TTH; however, due to the lack of attention, literature involving bibliometric analysis is sporadic. Therefore, this study aimed to explore the current hotspots and future directions of the TTH field by bibliometric analysis.

**Methods:**

By using CiteSpace and VOSviewer, literature regarding TTH between 2002 and 2021 from the Web of Science database was summarized and extracted. Annual publication trend, the most productive countries/regions and institutions, distribution of categories, co-citation of journals and references, and co-occurrence of keywords were analyzed.

**Results:**

A total of 3,379 publications were included in the final visualization, indicating a stable trend in current research and a lack of breakthroughs over the past decades. These studies were mainly conducted in 120 countries/regions led by the United States and more than 600 institutions. Four eternal core themes were identified in TTH, including neurosciences, nursing, developmental psychology, and general/internal medicine. *Cephalalgia* ranked first, with the highest number of literature, and is the most influential journal in this area. Keyword analysis demonstrated that the similarities and differences between migraine and TTH, epidemiological studies, clinical double-blind trials, and potential populations have become key issues in the TTH field.

**Conclusion:**

TTH has received less attention and breakthroughs in the past 20 years. To promote coordinated development between regions to fight headaches, cooperation and exchanges between countries and institutions are essential in the future. Relevant studies about headaches in children and adolescents, inducing factors such as emotional triggers and sleep disorders, concomitant diseases, possible pathogenesis, and headache treatments, are in the spotlight in recent years. This study offers a powerful roadmap for further research in this field.

## Introduction

Headache is a critical factor in increasing the global burden of disease (1), and tension-type headache (TTH) occurs in 42% of the adult population with an active headache disorder (2), which is a major factor that affects the disability-adjusted life-years of young and middle-aged people (3). TTH is the most common form of primary headache (4); typically, pain is often described as a pressing or tightening sensation of mild-to-moderate intensity in the bilateral regions of the head (5, 6). Based on headache frequency, TTH can be divided into three subtypes, namely, infrequent episodic, frequent episodic, and chronic TTH; infrequent episodic TTH has little effect on individuals with rare medical services, whereas chronic TTH is often associated with work-related disability and difficult treatment (5, 7). The lifetime prevalence of TTH in the general population ranges from 30 to 78% (8), with a higher incidence in female than in male patients (9). TTH has a huge effect on emotions, daily work, and general living activities, which not only leads to impaired social activities (10), such as a considerable number of work absences (11), but also brings a heavy economic burden to society (12). The potential pathophysiology of TTH remains poorly understood, and previous studies have reported that peripheral and central mechanisms play an important role in this process (13). Multimodal management is tailored for each patient with TTH according to different clinical symptoms and may include various therapies such as pharmacotherapy, behavioral therapies, and healthy lifestyle habits (5). Despite the widespread prevalence and considerable disability of TTH, a more comprehensive bibliometric analysis remains unavailable due to the lack of attention in this area.

To indicate critical issues in future studies, bibliometric analysis can reveal the current research status in a given field based on statistical and mathematical methods of publications (14). By using computerized analytic techniques, bibliometric analysis can estimate the most influential authors, journals, countries, departments, and institutions in a research area to identify publications that have influenced clinical practice and developed research ideas (15–17). The bibliometric analysis software, such as CiteSpace and VOSviewer, typically use scientific publications as input and generate an interactive visual network for statistical analysis (18). CiteSpace is a Java application that provides a progressive visual exploration of highly cited publications, frontier development in the current area, and emerging trends of research topics through knowledge discovery in bibliographic databases (19, 20). VOSviewer constructs bibliometric maps of influential authors, journals, and keywords in co-citation or co-occurrence analysis, with powerful functions and a user-friendly interface (21). These software have become important scientific mapping tools in the medical area, illuminating development trends and forecasts research outlook in a given field (22). Using the CiteSpace and VOSviewer software, this study was conducted to uncover the valuable insights and explore research hotspots of TTH over the past 20 years.

## Materials and methods

### Data collection

Citation data were retrieved from the Science Citation Index Expanded (SCI-expanded) of the Web of Science Core Collection database as of 17 January 2022. Considering the tremendous development of TTH in recent years, the search keyword was set to “tension-type headache” with a time span of 20 years from 2002 to 2021. Full records and cited references of publications were downloaded directly from the database, and document types were limited to articles or reviews.

### Data analysis

CiteSpace 5.8 R3 was adopted to generate visualization analysis and characteristic mapping, including a cooperation map of countries/regions and institutions, distribution of categories, number of citations, and keyword analysis. VOSviewer 1.6.17 was used to optimize unaesthetic diagrams. Moreover, the latest H-index, SCImago Institutions and Journal Rank, and Impact Factor were added for a clear and integrated analysis. ArcGIS 10.8 was applied to examine the national distribution of publications.

## Results

**Figure 1:**
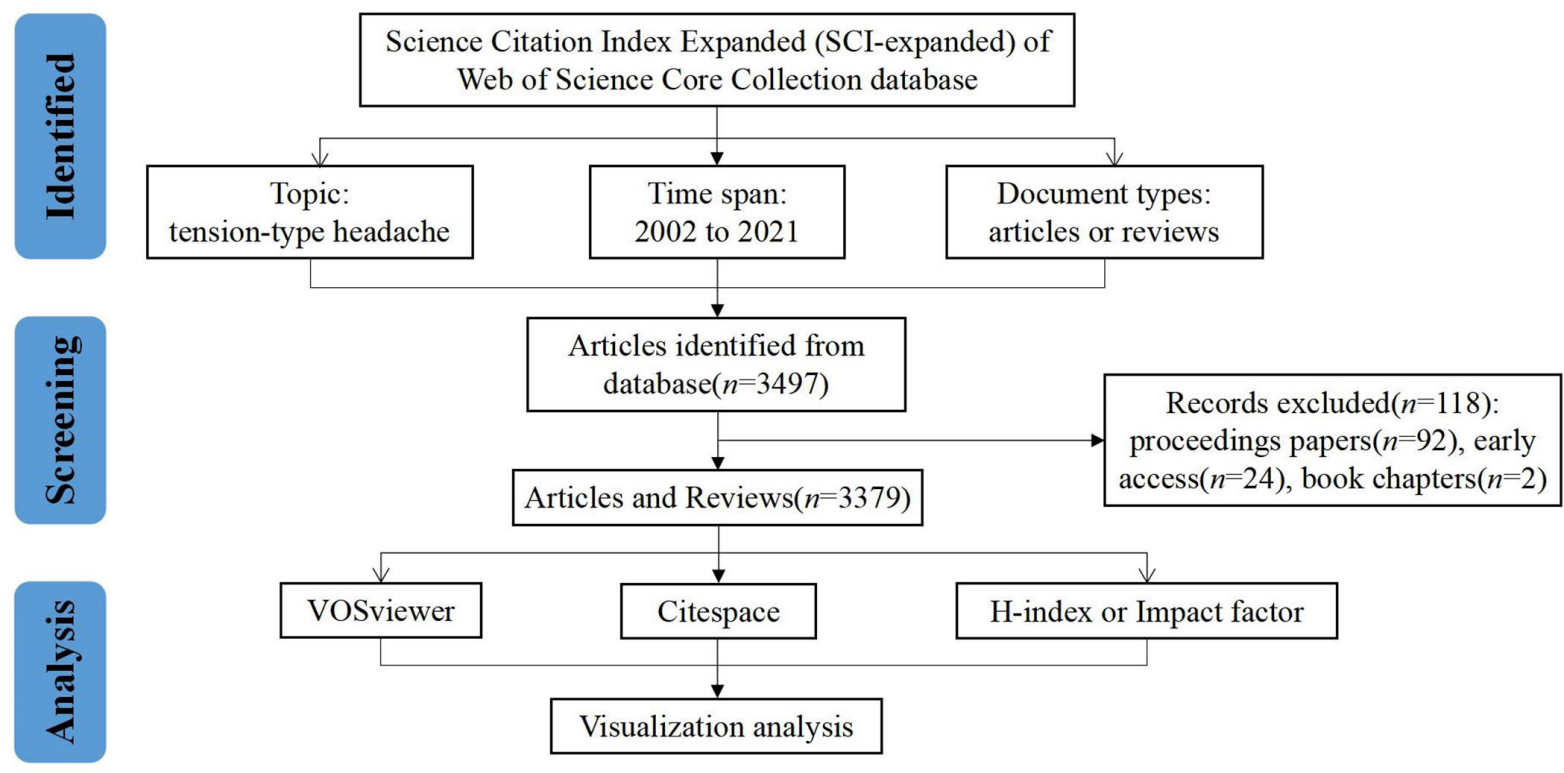
Flowchart of literature selection.

A total of 3,497 records were extracted from the database and 118 irrelevant articles were excluded, including 92 proceedings papers, 24 early access, and two book chapters. The remaining 3,379 records were exported for visualization analysis, of which 2,672 articles accounted for 79.08% of the total, followed by 707 reviews (20.92%). The retrieval process is presented in Figure 1.

### Annual publication outputs

**Figure 2:**
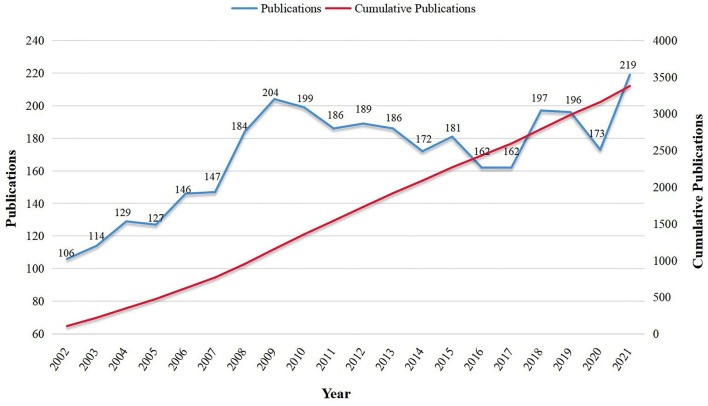
Publication trend of tension-type headache over the past 20 years.

The number of articles published in a specific period directly reflects the development trend of research in this field. As shown in Figure 2, despite the overall upward trend, publication growth on the topic of TTH can be divided into two periods, a rapid growth phase in the first decade and a slow phase in the second decade. From 2002 to 2009, publication outputs increased steadily year by year, but the volume fluctuated greatly from 2010 to 2021.

### Cooperation map of countries/regions

**Figure 3:**
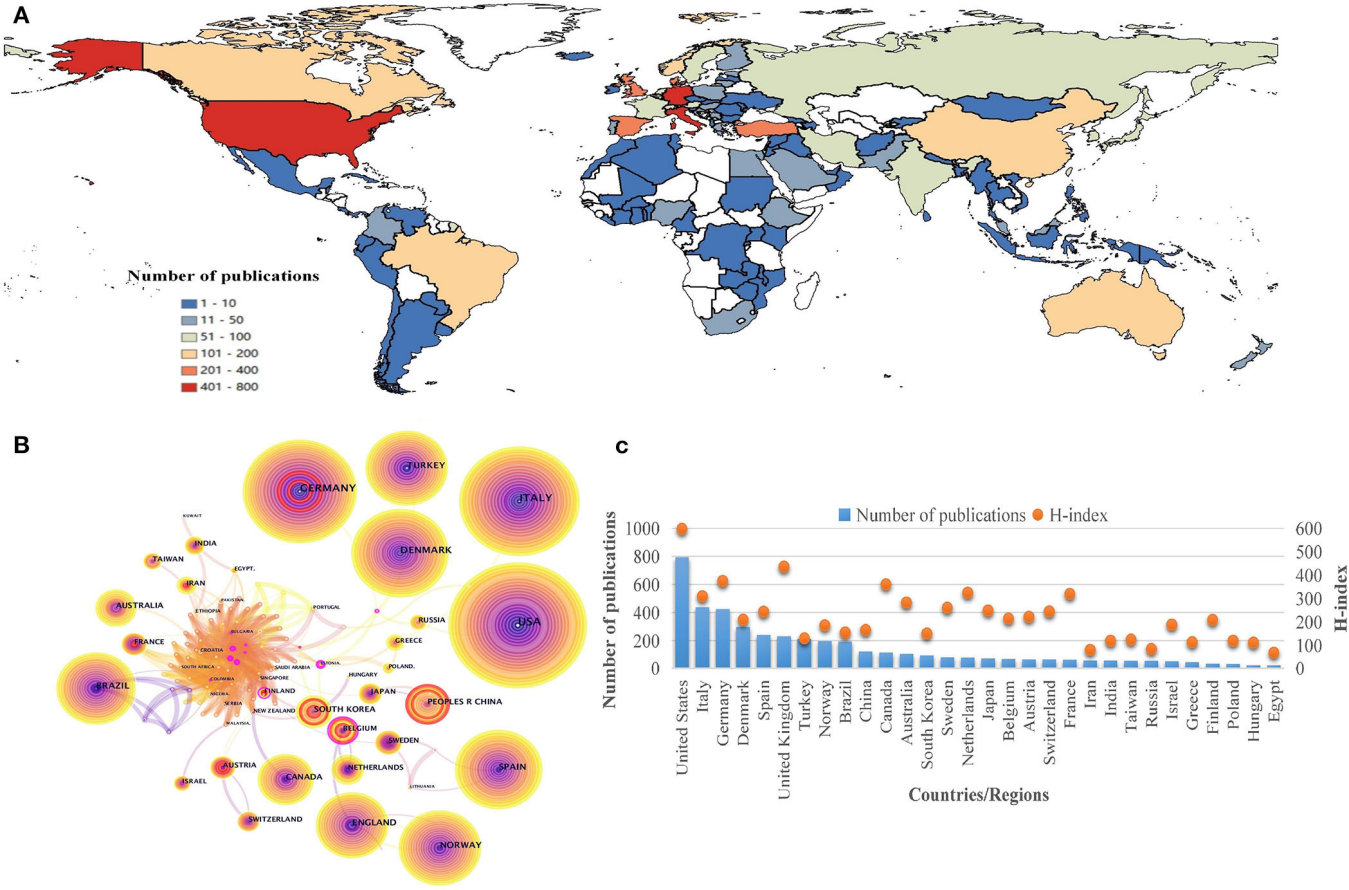
Geographical distribution and cooperation map of countries/regions in tension-type headache: **(A)** geographical heat map of publications; **(B)** co-occurrence network of countries/regions; **(C)** number of publications and H-index of countries/regions.

TTH research has been conducted in 120 countries/regions over the past 20 years (Figure 3A). In North America, the United States ranked first with 794 articles, followed by Italy and Germany in Europe with 439 and 426 publications, respectively. These countries contributed over one-third of the total number of publications and were labeled as three central research forces in this area. Denmark, the United Kingdom, and Spain in Europe, as well as Turkey in Asia, with more than 200 publications, will have great development space in the future. Besides, 76 countries (63.33% of the total) have <10 articles since 2002, and many countries/regions are still blank in this area.

Nodes in CiteSpace were selected to obtain the cooperation network map of countries/regions (Figure 3B). The node size represents the overall number of publications, and the lines between them indicate the cooperation relationship. Red nodes manifest the frequency burst, while pink rimmed nodes refer to those with high centrality, highlighting the importance of nodes in network (23). Germany, China, Belgium, South Korea, Sweden, and Austria have seen an increase in the number of publications over a certain period, speculating that they may have made innovative discoveries in TTH research. The H-index is a valuable indicator aimed at evaluating scientific influence of disciplines. Compared with other indexes, H-index can be more accurate in predicting future research achievement (24). By searching the H-index of the neurology field (Figure 3C), the United States has the highest score of 598, identifying its obvious advantage and outstanding contribution to TTH. The United Kingdom (436), Germany (375), and Canada (361) also made significant achievements in this area.

### Cooperation map of institutions

**Figure 4:**
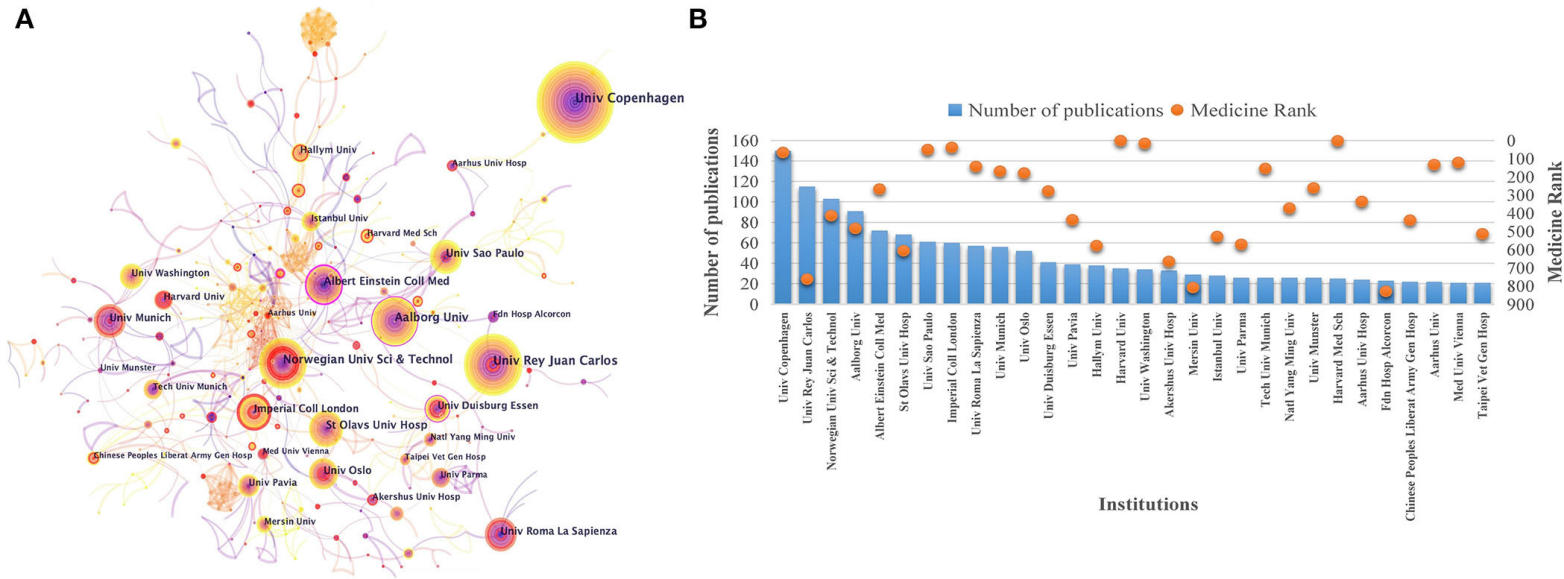
Cooperation maps of institutions in tension-type headache: **(A)** co-occurrence network of institutions; **(B)** number of publications and SCImago rank of institutions.

More than 600 institutions have explored TTH research over the past 20 years (Figure 4A). Among them, the University of Copenhagen in Denmark ranked first with 150 articles published, indicating its strong scientific ability in this area. The Universidad Rey Juan Carlos in Spain (115), Norwegian University of Science and Technology in Norway (103), and Aalborg University in Denmark (91) have also made significant contributions. Besides, the Universidade de São Paulo in Brazil, Imperial College London in the United Kingdom, and Universita Degli Studi di Roma La Sapienza in Italy have experienced a rapid increase in a certain period, indicating a breakthrough in these institutions. The SCImago Institutions Rankings in Medicine provide data references for scientific impact of institutions (25). Harvard University (1), Harvard Medical School (2), and the University of Washington (16) in the United States, as well as Medizinische Universitat Wien in Austria (121), have made vital contributions to TTH research field (Figure 4B).

### Distribution of categories

**Figure 5:**
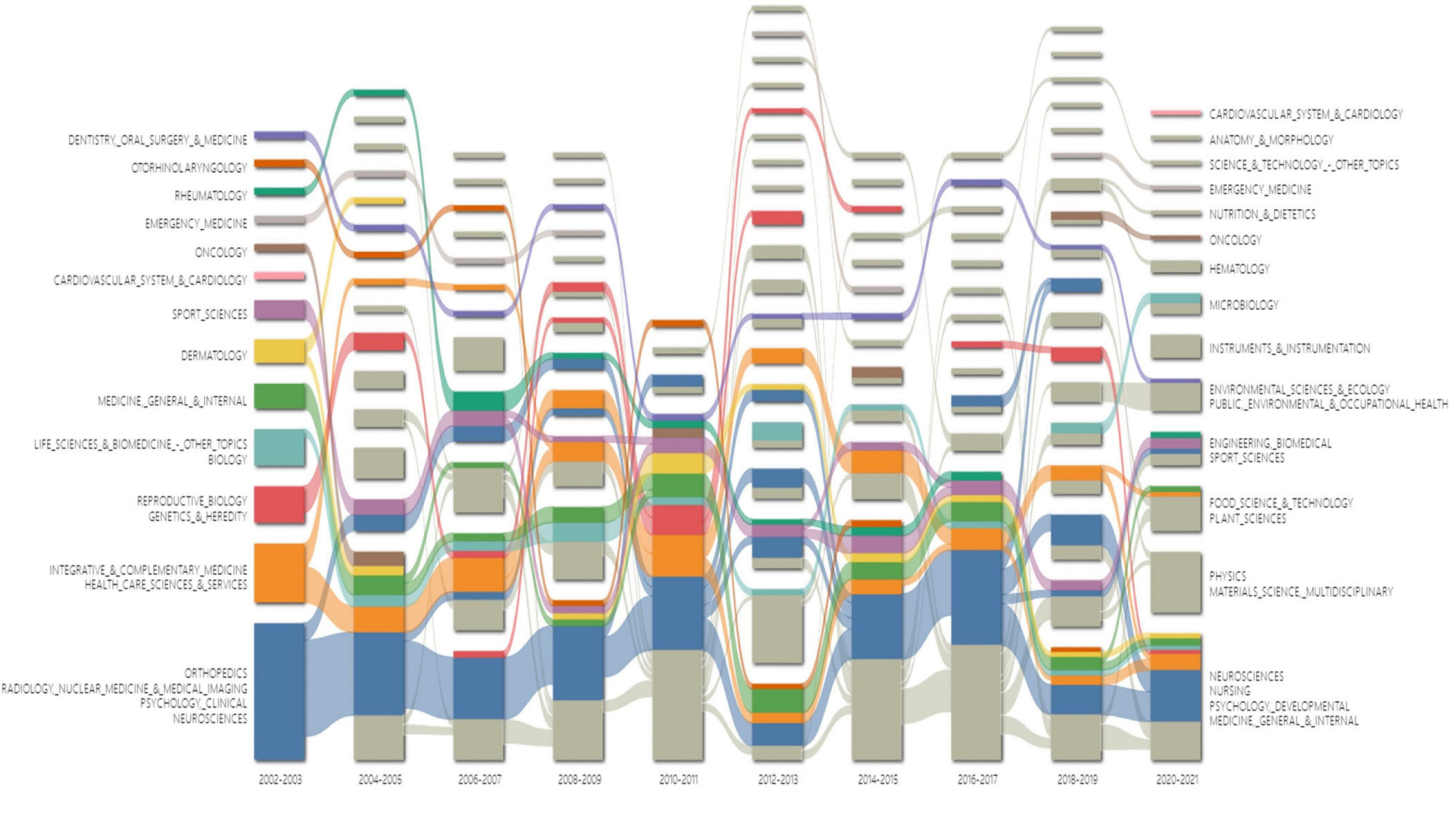
Alluvial diagram of categories in tension-type headache during 2002–2021.

To visualize changes in interdisciplinary situations in the TTH field, the alluvial diagram was adopted to highlight and summarize the structural variation of Web of Science categories (Figure 5). The network is displayed as vertical stacks connected by streamlines that joint modules containing the same nodes. The height changes of the streamlines are proportional to the aggregated flow of nodes in the connected modules (26). Structural changes from one period to the next are represented by mergers and divergences, with different categories plotted in different colors. The interdisciplinary change has been ongoing from 13 modules in 2002–2003 to 14 modules in 2020–2021. The modules represented by neurosciences, healthcare sciences/services, genetics/heredity, biology, and general/internal medicine are gradually summarized into the four eternal core themes led by neurosciences, nursing, developmental psychology, and general/internal medicine. Besides, physics, plant sciences, and public environmental/occupational health as newly emerging themes are in the spotlight in recent years.

The statistical indicators of the top 15 categories are listed in Table 1, indicating that TTH is a multidisciplinary research area. Neurosciences and neurology ranked first with 2,339 articles published since 2002, followed by clinical neurology with 1,296 articles. Although the number of publications is much lower than the top categories, public environmental/occupational health, and pediatrics have high betweenness centrality, indicating their critical position in this field.

**Table 1:** Top 15 categories in the tension-type headache field.

**Rank**	**Category**	**Publications**	**Centrality**	**Starting year**
1	Neurosciences and neurology	2,339	0.48	2002
2	Clinical neurology	1,296	0.06	2002
3	General and internal medicine	275	0.08	2002
4	Anesthesiology	223	0	2002
5	Psychiatry	166	0.09	2002
6	Pediatrics	125	0.22	2002
7	Pharmacology and pharmacy	125	0.04	2002
8	Rehabilitation	118	0.02	2002
9	Neurosciences	101	0.13	2002
10	Dentistry, Oral surgery and medicine	79	0.02	2002
11	Integrative and complementary medicine	74	0.01	2002
12	Public environmental and occupational health	51	0.31	2005
13	Sport sciences	48	0.06	2002
14	Research and experimental medicine	46	0.10	2002
15	Science and technology-other topics	45	0	2009

### Journal and co-cited journals

A total of 620 journals reported research in TTH, and the top 15 are presented in Table 2. *Cephalalgia* ranks first with 419 publications (12.40%), followed by *Headache* (364, 10.77%) and *Journal of Headache and Pain* (264, 7.81%). Among the top 15 academic journals, five are from the United States, four from the United Kingdom, and two from Italy, showing a strong research foundation in these countries. Six journals have an impact factor >5, including *Neurology* (11.800), *Journal of Headache and Pain* (8.588), *Pain* (7.926), *European Journal of Neurology* (6.288), *Cephalalgia* (6.075), and *Headache* (5.311). SCImago Journal Rankings measure the scientific impact, influence, and prestige of journals based on the average number of weighted citations (27). Eight journals (*Cephalalgia, Headache, Journal of Headache and Pain, Pain, Neurology, European Journal of Pain, European Journal of Neurology*, and *Frontiers in Neurology*) are considered of Q1 quality with far-reaching development prospects.

**Table 2:** Top 15 journals in the tension-type headache field.

**Rank**	**Journal**	**Publications (%)**	**Country/region**	**Impact factor (2021)**	**SCImago journal rank**	**Quartiles**	**H-index**
1	Cephalalgia	419 (12.40%)	The United Kingdom	6.075	1.805	Q1	131
2	Headache	364 (10.77%)	The United Kingdom	5.311	1.472	Q1	126
3	Journal of Headache and Pain	264 (7.81%)	Italy	8.588	1.924	Q1	70
4	Neurological Sciences	113 (3.34%)	Italy	3.830	0.847	Q2	76
5	Current Pain and Headache Reports	92 (2.72%)	The United States	3.904	0.621	Q3	68
6	Pain	65 (1.92%)	The United States	7.926	2.135	Q1	269
7	Arquivos de Neuro-Psiquiatria	48 (1.42%)	Brazil	2.035	0.427	Q3	52
8	Clinical Journal of Pain	41 (1.21%)	The United States	3.423	0.876	Q2	130
9	Neurology	40 (1.18%)	The United States	11.800	2.587	Q1	378
10	European Journal of Pain	40 (1.18%)	The United States	3.651	0.839	Q1	114
11	European Journal of Neurology	40 (1.18%)	The United Kingdom	6.288	1.662	Q1	130
12	Frontiers in Neurology	36 (1.07%)	Switzerland	4.086	1.027	Q1	80
13	Revista de Neurologia	33 (0.98%)	Spain	1.235	0.297	Q3	43
14	Schmerz	24 (0.71%)	Germany	1.629	0.262	Q4	43
15	BMC Neurology	24 (0.71%)	The United Kingdom	2.903	0.772	Q2	82

**Figure 6:**
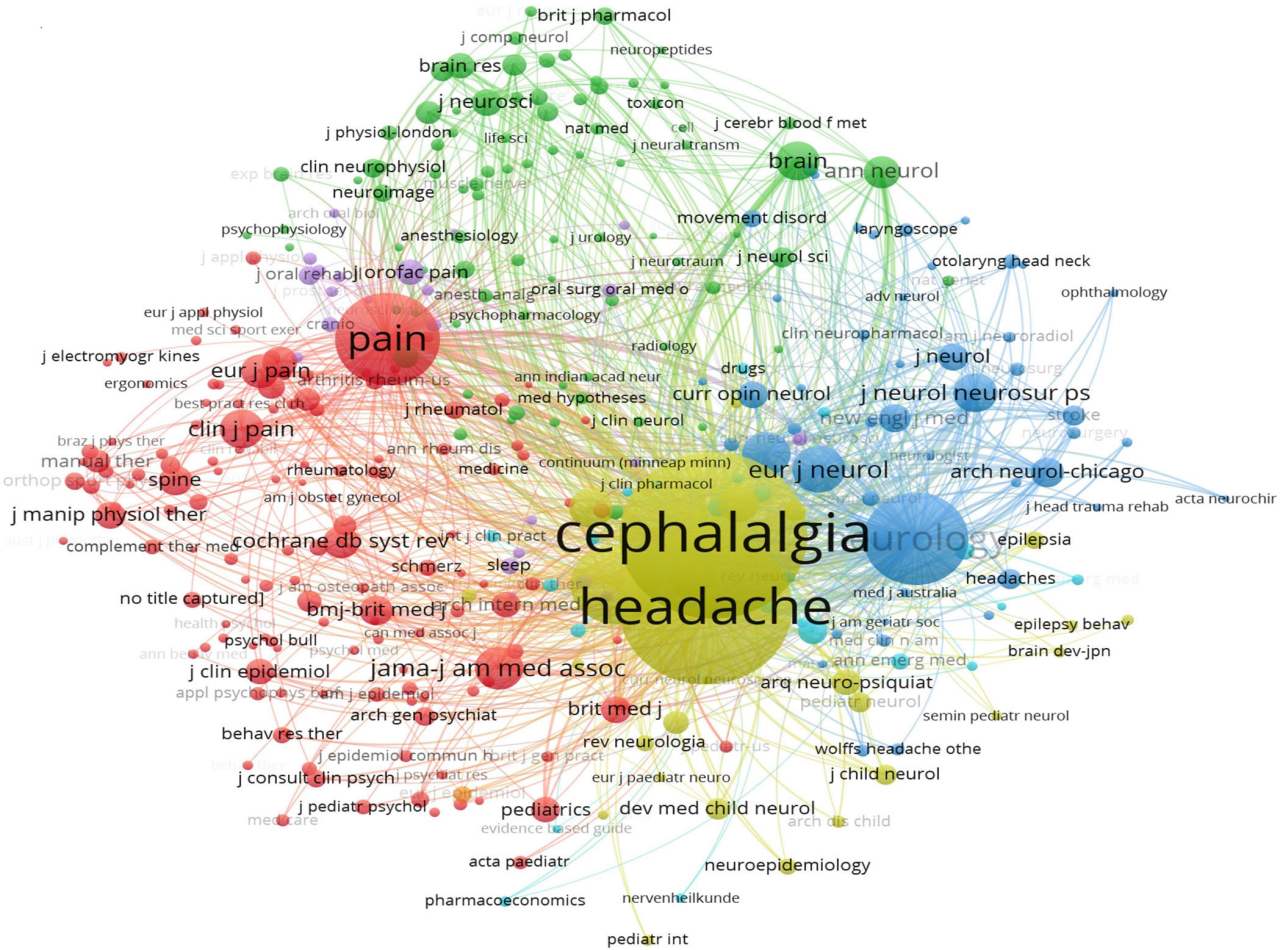
Co-cited journals in tension-type headache.

The influence of journals in a particular research field depends on the number of co-citations, i.e., when two articles published in different journals were cited in a third article of another journal. The analysis was performed in VOSviewer (Figure 6). The clusters were generated based on citation links and were marked with different colors. The size of the node indicates the co-citated number of journals, and the lines between them represent the co-citation relationship. *Cephalalgia*, hosted by SAGE Publications Ltd in the United Kingdom, is the most influential journal with 3,345 citations, indicating the highest recognition and authority in the TTH area. Compared with journal analysis, in the top co-cited journals, six of 15 have an impact factor >10, including *Lancet* (202.731), *JAMA – Journal of the American Medical Association* (157.335), *Lancet Neurology* (59.935), *Brain* (15.255), *Journal of Neurology, Neurosurgery and Psychiatry* (13.654), and *Neurology* (11.800), which demonstrated that the theoretical sources of TTH articles are of high quality.

### Co-cited references

A total of 1,420 co-cited references were analyzed, and the top 15 are listed in Table 3. The review “Migraine pathophysiology and its clinical implications” (28) in *Cephalalgia* ranked first with 374 citations. Other references were cited between 40 and 300 times. In addition, topics covered by highly co-cited references include epidemiological studies, clinical guidelines, and progress reports. The top 15 co-cited references were all published in journals with an impact factor >5, and five were in top journals, including two in *Lancet* (202.731) and three in *Lancet Neurology* (59.935).

**Table 3:** Top 15 co-cited references in the tension-type headache field.

**Rank**	**Title**	**Citation**	**Year**	**Journal**	**Country/region**	**Impact factor (2021)**
1	Migraine pathophysiology and its clinical implications	374	2004	Cephalalgia	The United Kingdom	6.075
2	The international classification of headache disorders, 3rd edition (beta version)	240	2013	Cephalalgia	The United Kingdom	6.075
3	The global burden of headache: a documentation of headache prevalence and disability worldwide	141	2007	Cephalalgia	The United Kingdom	6.075
4	Pain Global, regional, and national burden of migraine and tension-type headache, 1990–2016: a systematic analysis for the Global Burden of Disease Study 2016	105	2018	Lancet Neurology	The United Kingdom	59.935
5	New appendix criteria open for a broader concept of chronic migraine	90	2006	Cephalalgia	The United Kingdom	6.075
6	Years lived with disability (YLDs) for 1,160 sequelae of 289 diseases and injuries 1990-2010: a systematic analysis for the Global Burden of Disease Study 2010	85	2012	Lancet	The United Kingdom	202.731
7	Prevalence of neck pain in migraine and tension-type headache: a population study	67	2015	Cephalalgia	The United Kingdom	6.075
8	Global, regional, and national incidence, prevalence, and years lived with disability for 301 acute and chronic diseases and injuries in 188 countries, 1990-2013: a systematic analysis for the Global Burden of Disease Study 2013	53	2015	Lancet	The United Kingdom	202.731
9	EFNS guideline on the treatment of tension-type headache - report of an EFNS task force	52	2010	European Journal of Neurology	The United Kingdom	6.288
10	Tension-type headache: the most common, but also the most neglected, headache disorder	49	2006	Current Opinion in Neurology	The United States	6.283
11	Headache Classification Committee of the International Headache Society (IHS) the international classification of headache disorders, 3rd edition	47	2018	Cephalalgia	The United Kingdom	6.075
12	Has the prevalence of migraine and tension-type headache changed over a 12-year period? A Danish population survey	47	2005	European Journal of Epidemiology	Netherlands	12.434
13	Tension-type headache: current research and clinical management	45	2008	Lancet Neurology	The United Kingdom	59.935
14	The prevalence and burden of primary headaches in China: a population-based door-to-door survey	45	2012	Headache	The United Kingdom	5.311
15	Epidemiology and comorbidity of headache	44	2008	Lancet Neurology	The United Kingdom	59.935

**Figure 7:**
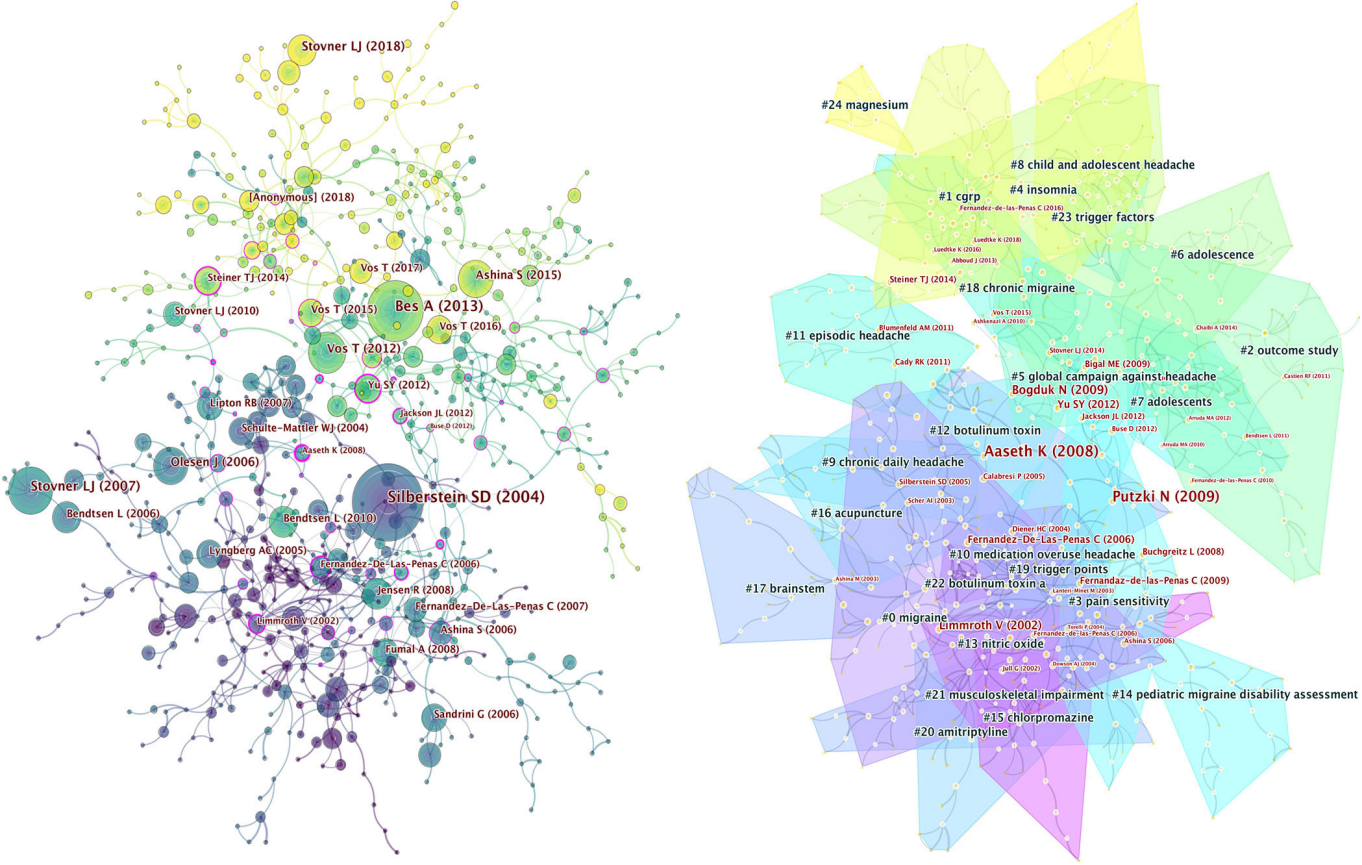
Co-cited networks and cluster diagrams of references in tension-type headache.

Co-cited network and cluster analysis of references was conducted in CiteSpace (Figure 7). Color corresponds to the year in which the publications appeared, and the depth of the color represents time. All references were divided into 24 clusters with active co-citation relationships between them, including pain sensitivity (#3), chronic daily headache (#9), acupuncture (#16), trigger points (#19), botulinum toxin a (#22), and amitriptyline (#21). Clusters located in the upper part of the figure are lighter in color, indicating that calcitonin gene-related peptide (CGRP), insomnia, child and adolescent headache, trigger factors, and magnesium are research hotspots in recent years.

### Keyword analysis

**Figure 8:**
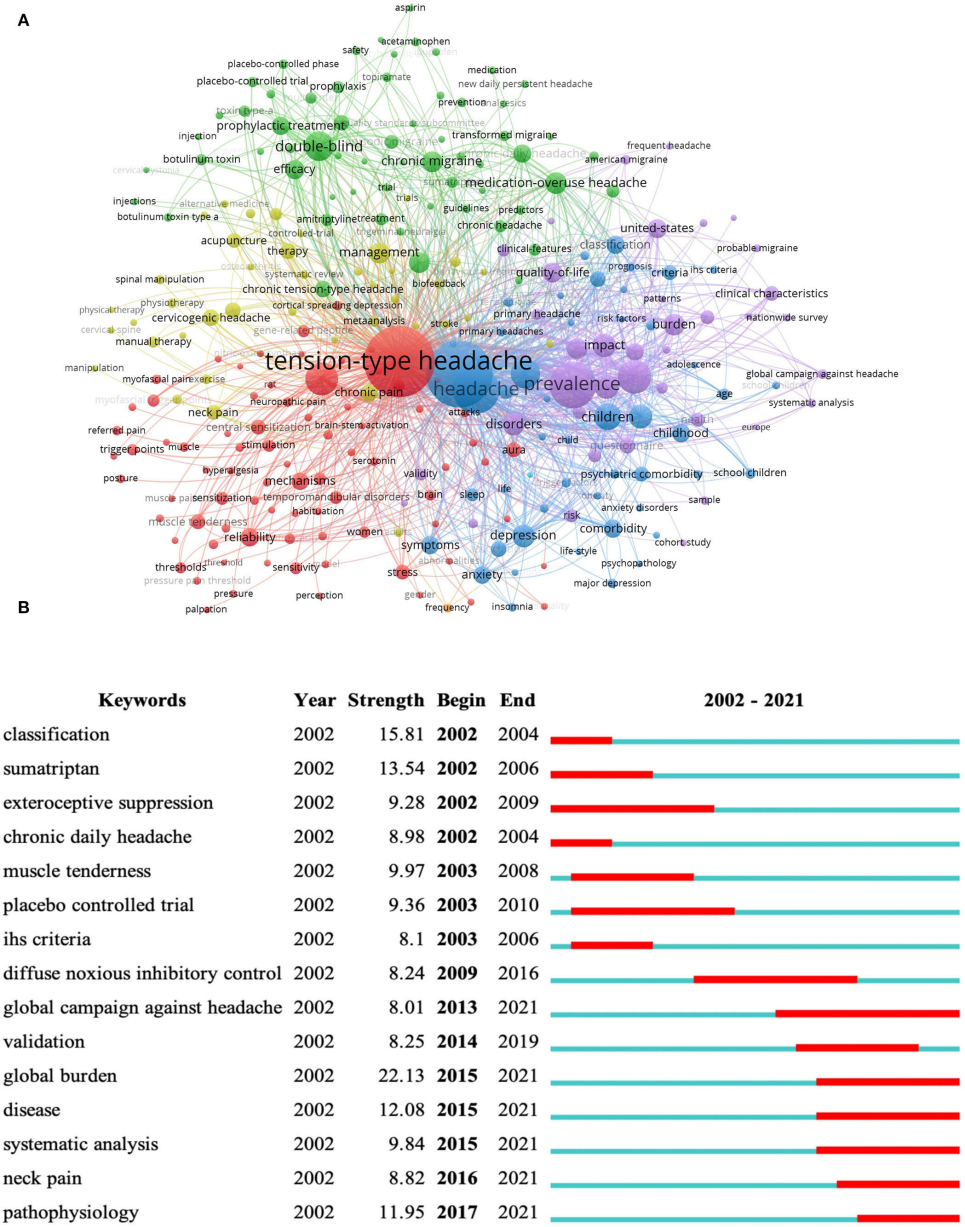
Keyword analyses in tension-type headache: **(A)** co-occurrence network of keywords; **(B)** top 15 keywords with the strongest citation bursts.

Keyword analysis provided a typical overview of research topics, reflecting the hotspots and directions in a particular field. The network mapping of high-frequency keywords was performed in VOSviewer (Figure 8A). The keyword “tension-type headache” ranked first with 2,171 times, which is consistent with the research topic. In addition, the top high-frequency keywords included migraine (1,042), prevalence (850), double-blind (398), epidemiology (393), and population (377), leading to speculations that identifying similarities and differences between migraine and TTH, strengthening epidemiological research, conducting clinical double-blind trials, and searching potential population have become key issues in the TTH area.

Keywords with citation bursts indicate the rapid increase in research topics in a given period and highlight the possible research priorities in the near future. The top 15 with the strongest citation bursts were identified in CiteSpace (Figure 8B). Keywords such as classification (15.81, 2002–2004), muscle tenderness (9.97, 2003–2008), exteroceptive suppression (9.28, 2002–2009), and International Headache Society (IHS) criteria (8.1, 2003–2006) inferred that the classification criteria of the subtypes and mechanism of peripheral stimulation were the hotspots of TTH in the first 10 years; while keywords like global burden (22.13, 2015–2021), pathophysiology (11.95, 2017–2021), diffuse noxious inhibitory control (8.24, 2009–2016), and global campaign against headache (8.01, 2013–2021) manifested that the globalization of TTH damage and pathogenesis of central sensitization have become the focus of current research in the TTH field.

**Figure 9:**
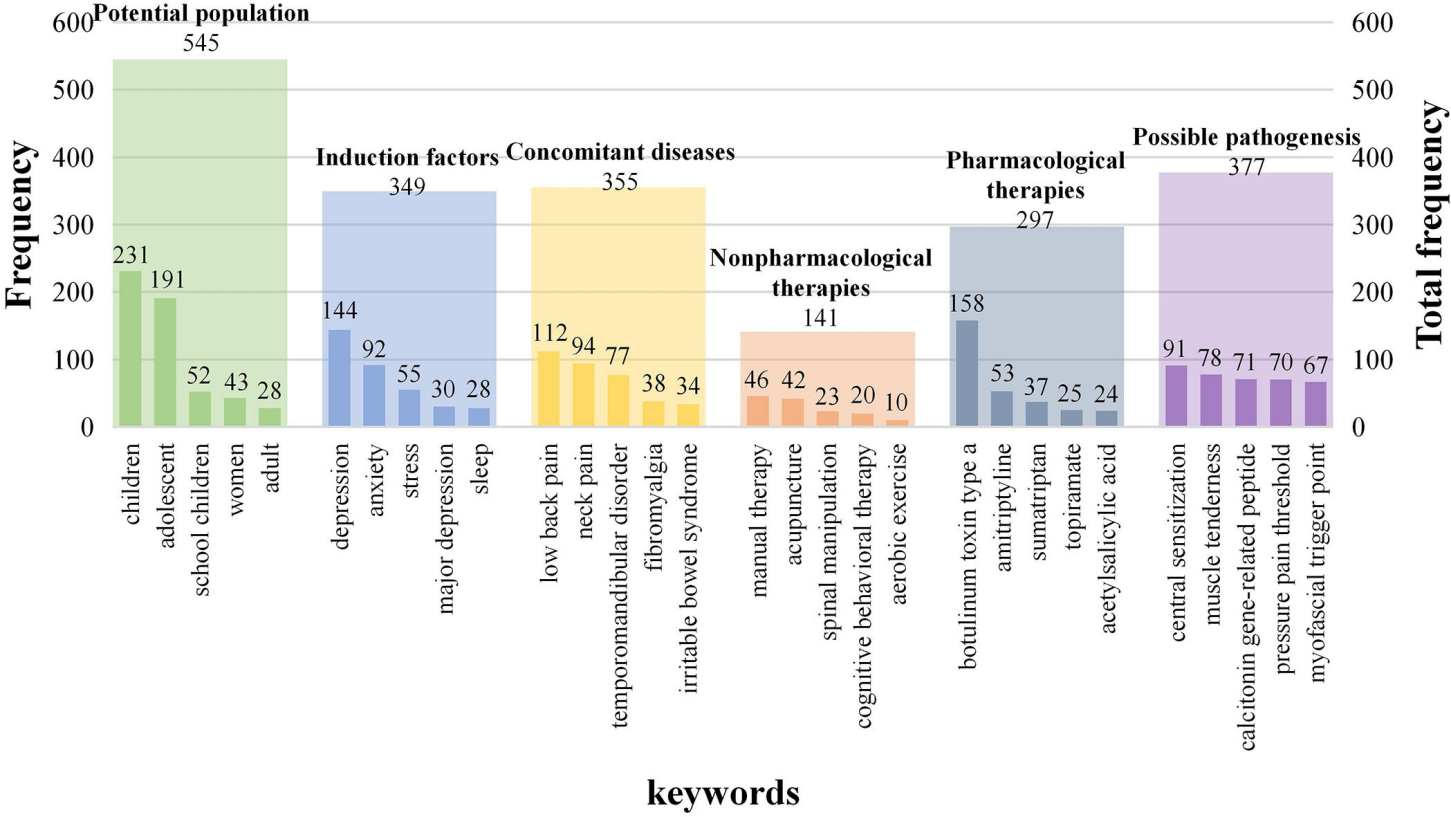
Keyword analyses in five dimensions.

Besides, to better illustrate the current hotspots and future directions, this study conducted manual statistics based on the top five keywords in each classification, including potential population, induction factors, concomitant diseases, possible pathogenesis, and clinical therapies (Figure 9).

## Discussion

### General information

As regards annual publication outputs, the trend of literature published from 2002 to 2021 went through two growth stages, publications in 2021 were just 2.07 times over 2002, manifesting a stable trend in current research and a lack of breakthroughs over the past decades. The finding that most patients with TTH rarely seek medical help compared with migraines or other type of headaches is probably the main reason that this area of research received less attention from health authorities, clinical researchers, or professional pharmacologists (5, 29); and as a result, TTH has made little progress since the early 2010s.

On the cooperation networks of countries and institutions, although TTH studies have been conducted in 120 countries led by the United States and more than 600 institutions, most regions still lacked study data in the area. This phenomenon can be explained from two aspects. The social perception of headache damage is at low level in developing countries, leading to less investment in research funds. In addition, due to brain drain, backward technology, and lack of frontiers, the majority of publications on headache disorders still come from developed countries (30–32). Such uneven development among regions revealed that the negative effects of TTH have not received widespread attention. Although coordinated development between regions and institutions remains inadequate, the current cooperation between countries indicated an active relationship. Lifting The Burden, a UK-registered charity organization, established an official relationship with the World Health Organization to conduct the Global Campaign against Headache (33), which, to a certain extent, promotes the mutual collaboration between countries and institutions (34).

TTH is a multidisciplinary development research field. Previous studies identified that TTH is at a relatively high risk of developing ischemic stroke (35), health nursing delivering treatments efficiently to patients, and will substantially reduce the ill-health burden of headache (36); similarly, emotional factors are potential causes of unfavorable prognosis and outcomes from the preventive treatment in headache (37). Population-based studies have manifested that anxiety and depression are more common in patients with TTH (38, 39), which lead to an increase in headache frequency and play a relevant role in the association between pain interference and burden (40). Therefore, psychological therapies can reduce emotional burden and relieve headache symptoms in patients. This explains the critical role of nursing and developmental psychology in the TTH field. In addition, the prevalence of TTH among children and adolescents ranges from 10 to 25% (41), resulting in impaired daily activities and absence from school (42). Understanding the mechanism of headache and finding specific drugs for treatment may help reduce the incidence of pain and the occurrence of concomitant diseases in children and adolescents. Besides, stress-related headaches usually lead to inefficient work during attacks (43), thus a leisurely work environment probably contributes to the alleviation. Although the number of publications is much lower than the top categories, public environmental/occupational health and pediatrics have also attracted widespread attention in TTH.

*Cephalalgia* ranks first with most literature and is the most influential journal in the area, with the most citation reviews of migraine pathophysiology. Considering that the pathogenesis of TTH remains unclear, most researchers tend to endorse peripheral and central sensitization, which is independent of headache types, demonstrating the clinical similarity of various forms of chronic headache (44). In other words, the mechanism of migraine may have certain reference in TTH, which is perhaps the main reason that such studies are most cited in the TTH area.

### Hotspots and frontiers

In view of the current research situation of the TTH field, the following suggestions are provided for future research hotspots based on reference and keyword analysis.

*About the potential population*. Although the prevalence of TTH assorted between countries and regions due to differences in study methods and demographic characteristics (45), the peak arose in adults aged 35–39 years, and the percentage of disability-adjusted life-years was high in children and adolescents (32). Headache has been a common grievance in childhood, and younger children seem to be more sensitive to pain stimulation (46). In spite of the symptoms of TTH being not different from those in adults, the duration of attack can be shorter and more variable (47). Headache in children and adolescents should be the priority of future research; in addition to the tremendous impact on daily life and learning, sufficient attention should be paid to whether severe and frequent headaches over a long period of time can lead to mental impairment in children or even to various diseases caused by pain sensitivity in adulthood.

*About the induction factors*. The role of psychological factors associated with TTH has long been the focus of headache investigation (48). The high depression or anxiety score at baseline was strongly associated with a substantially increased risk of headache (49). Besides, individuals with TTH often complain of sleep disturbances. Previous studies have demonstrated that insomnia, insufficient sleep, and poor sleep quality can result in TTH, with an increased risk of chronic form (50). The association between headaches and emotions or sleep may be bidirectional, but the potential effects are unclear, and whether targeted preventive measures have definite benefits for both psychological and sleep factors need to be further explored.

*About the concomitant diseases*. The characteristics of temporomandibular disorders are often accompanied by symptoms that are not directly related to the functioning of joints, such as TTH (51). The positive intersections had been reported in previous studies, namely, resulting in higher headache frequency and the Headache Impact Test (HIT-6) scores, and the occurrence of chronic headache (52). Fibromyalgia is a chronic pain syndrome of unknown etiology that appears to be prevalent in patients with TTH (53). When a headache occurs, its severity is associated with increased fibromyalgia symptoms, including frequent headaches, anxiety, pericranial tenderness, physical performance, and sleep disturbances (54, 55). Central sensitization is an important part of the abnormal endogenous pain regulation and somatic hypersensitivity, which is highly similar to the underlying pathogenesis of headache, indicating that TTH is the main cause of comorbidities. However, somatic pain such as back and neck pain in some individuals may be strongly associated with a lower pericranial pressure pain threshold, leading to a view that central sensitization may be a consequence of comorbidity rather than a cause (56). Clarifying the relationship between TTH and comorbidities may help understand the pathogenesis. The collaboration of a multidisciplinary clinical team may have significant benefits for the treatment of TTH, which should be the focus of future research.

*About the non-pharmacological therapies*. Non-drug treatments for TTH are widely used as a supplement or substitute to medical treatment. Manual therapy is a physical treatment used by practitioners to treat musculoskeletal pain and disability (57). Previous analysis manifested that despite the low certainty of evidence, manual therapy has potential positive effects on headache frequency and quality of life (58). Besides, acupuncture, which originated in China, is a therapy that involves inserting thin needles into the skin at specific points; it is used in many countries to treat headaches. Given its low side effects and high patient acceptance, acupuncture demonstrated significant improvement in pain measures, disability index, quality of life, and psychological status (59, 60). The Cochrane Database of Systematic Reviews acknowledged the potential of acupuncture as a valuable therapy for frequent episodic or chronic TTH (61). In addition, cognitive behavioral therapy is an effective strategy for reducing headache episodes over time (62). Previous studies have demonstrated that cognitive behavioral therapy is associated with a better reduction in pain intensity, mood, headache-related disability, and quality of life (63), forming a valid option for the prevention of headaches at a young age (41, 64). Nevertheless, because of the inevitable unblinded method and detection bias in experimental process, the intervention effect on headache might be overestimated. Therefore, large and high-quality studies are needed to clarify the efficacy.

*About the pharmacological therapies*. The dominant opinion on the underlying mechanism of botulinum toxin is to inhibit the release of peripheral neurotransmitters or inflammatory mediators, with a secondary effect on the central sensitization, preventing the progression to chronic TTH (65). Several studies have identified that chronic TTH could benefit from the use of botulinum toxin-based on careful patient selection, specific injection patterns, and clear treatment targets (66). Although there are studies indicated that compared with placebo, botulinum toxin was not associated with chronic TTH (67). Such contradictory findings are probably related to their exclusion criteria, and further studies are warranted to determine the efficacy. In the preventive treatment of TTH, the tricyclic antidepressant amitriptyline is the only drug that has proven to be effective, leading to rapid reduction in headache activity, use of analgesic medications, and headache-related disability (68). However, multiple side effects caused by amitriptyline such as dry mouth, drowsiness, dizziness, obstipation, and weight gain may hamper the treatment effectiveness (69). Acetylsalicylic acid (aspirin) is among the most commonly used drugs for the management of pain symptomatologies (70). In frequent episodic TTH, aspirin provided some effects in adults with acute headaches of moderate or severe intensity. Nevertheless, owing to the limited quantity and quality of evidence, such benefit should be interpreted with caution in clinical practice (71). Therefore, the treatment of TTH is still the focus and difficulty of clinical research, and there is an urgent need to develop specific pharmacological management with higher efficacy and fewer side effects.

*About the possible pathogenesis*. The pathogenesis of TTH mainly focuses on peripheral and central sensitization, but the specific mode of action remains unclear. CGRP is considered as a pain-signaling neuropeptide and a potent vasodilator (72). Previous studies have indicated that the plasma levels of CGRP are normal in patients with chronic TTH and are not associated with the headache status (73). However, considering the pathophysiological importance of CGRP, whether TTH can be treated with monoclonal antibodies targeting CGRP or its receptor-like migraine requires various studies to elucidate this issue, and further research is needed to explore the differences between migraine and TTH.

### Limitations

Although CiteSpace and VOSviewer have become important mapping tools in the medical field, bibliometric analysis cannot replace system retrieval because of methodological limitations. First, the publications retrieved only included articles and reviews in the Web of Science database, ignoring contributions from other forms of publications. The limitation of the retrieval strategy may lead to the uncertainty of research conclusions. Second, bibliometric analyses generally focus on the influence of a publication rather than on the quality; the inclusion of low-quality articles may affect analysis results. Finally, a unified standard is still lacking in bibliometric analysis; different types of publications may have more appropriate research methods. Disease research should consider complicated factors, which cannot be quantified in simple diagrams and need to be discussed in future research.

## Conclusion

Despite the lack of attention and breakthroughs in the past decades, TTH has vital research value and broad development prospects and urgently needs cooperation and exchanges between countries and institutions. Relevant studies about headaches in children and adolescents, inducing factors such as emotional and sleep, concomitant diseases, and clinical management related to central sensitization, are in the spotlight in recent years, which are also the hotspot of future research.

## Data availability statement

The raw data supporting the conclusions of this article will be made available by the authors, without undue reservation.

## Author contributions

XF conceived and designed this study, retrieved the articles, collected the data, and drafted the manuscript. XF, GF, and LW analyzed the data and drew the maps. WS and YZ directed the research. All authors contributed to the article and approved the final manuscript.

## Funding

This research was supported by the Capital Health Research and Development of Special (No. 2020-2-4173), Scientific and Technological Innovation Project of China Academy of Chinese Medical Sciences (No. CI2021B006), Innovation Team and Talents Cultivation Program of National Administration of Traditional Chinese Medicine (No. ZYYCXTD-C-202007), and the National TCM Leading Personnel Support Program [NATCM Personnel and Education Department (2018)] (No. 12).

## Conflict of interest

The authors declare that the research was conducted in the absence of any commercial or financial relationships that could be construed as a potential conflict of interest.

## Publisher's note

All claims expressed in this article are solely those of the authors and do not necessarily represent those of their affiliated organizations, or those of the publisher, the editors and the reviewers. Any product that may be evaluated in this article, or claim that may be made by its manufacturer, is not guaranteed or endorsed by the publisher.
